# Online Adaptive MRI-Guided Radiotherapy for Primary Tumor and Lymph Node Boosting in Rectal Cancer

**DOI:** 10.3390/cancers15041009

**Published:** 2023-02-05

**Authors:** Chavelli M. Kensen, Anja Betgen, Lisa Wiersema, Femke P. Peters, Mutamba T. Kayembe, Corrie A. M. Marijnen, Uulke A. van der Heide, Tomas M. Janssen

**Affiliations:** 1 Department of Radiation Oncology, The Netherlands Cancer Institute, 1066 CX Amsterdam, The Netherlands; 2 Department of Scientific Administration, The Netherlands Cancer Institute, 1066 CX Amsterdam, The Netherlands

**Keywords:** rectal cancer, online adaptive radiotherapy, MRI-guided radiotherapy, intrafraction motion, interfraction motion, PTV margin, mesorectal lymph nodes

## Abstract

**Simple Summary:**

Improving clinical complete response (cCR) rates after neo-adjuvant (chemo)radiotherapy may facilitate organ sparing in intermediate-risk and locally advanced rectal cancer. Increasing the radiotherapy dose will possibly increase response rates. The potential of dose escalation in rectal cancer is limited by substantial PTV margins to accommodate inter- and intrafraction anatomical variation. Online adaptive MRI-guided radiotherapy offers good soft tissue contrast and the possibility to adapt the treatment to the daily anatomy. This approach has the potential to make dose escalation to multiple targets in rectal cancer feasible. With online adaptive MRI-guided radiotherapy, daily plan adaptation can be performed through the use of two different strategies. The purpose of this study was to characterize the motion and define the required treatment margins of the pathological mesorectal lymph nodes and the primary tumor for these two strategies and to study the effect of the anatomical location of the lymph nodes on the strategies.

**Abstract:**

The purpose of this study was to characterize the motion and define the required treatment margins of the pathological mesorectal lymph nodes (GTV_ln_) for two online adaptive MRI-guided strategies for sequential boosting. Secondly, we determine the margins required for the primary gross tumor volume (GTV_prim_). Twenty-eight patients treated on a 1.5T MR-Linac were included in the study. On T2-weighted images for adaptation (MRI_adapt_) before and verification after irradiation (MRI_post_) of five treatment fractions per patient, the GTV_ln_ and GTV_prim_ were delineated. With online adaptive MRI-guided radiotherapy, daily plan adaptation can be performed through the use of two different strategies. In an adapt-to-shape (ATS) workflow the interfraction motion is effectively corrected by redelineation and the only relevant motion is intrafraction motion, while in an adapt-to-position (ATP) workflow the margin (for GTV_ln_) is dominated by interfraction motion. The margin required for GTV_prim_ will be identical to the ATS workflow, assuming each fraction would be perfectly matched on GTV_prim_. The intrafraction motion was calculated between MRI_adapt_ and MRI_post_ for the GTV_ln_ and GTV_prim_ separately. The interfraction motion of the GTV_ln_ was calculated with respect to the position of GTV_prim_, assuming each fraction would be perfectly matched on GTV_prim_. PTV margins were calculated for each strategy using the Van Herk recipe. For GTV_ln_ we randomly sampled the original dataset 20 times, with each subset containing a single randomly selected lymph node for each patient. The resulting margins for ATS ranged between 3 and 4 mm (LR), 3 and 5 mm (CC) and 5 and 6 mm (AP) based on the 20 randomly sampled datasets for GTV_ln_. For ATP, the margins for GTV_ln_ were 10–12 mm in LR and AP and 16–19 mm in CC. The margins for ATS for GTV_prim_ were 1.7 mm (LR), 4.7 mm (CC) and 3.2 mm anterior and 5.6 mm posterior. Daily delineation using ATS of both target volumes results in the smallest margins and is therefore recommended for safe dose escalation to the primary tumor and lymph nodes.

## 1. Introduction

Neo-adjuvant (chemo)radiotherapy reduces the risk of local recurrence and downstages the tumor in patients with intermediate-risk and locally advanced rectal cancer (LARC) [1]. Studies by Habr-Gama et al. [2,3,4] showed the feasibility of active surveillance instead of surgery for patients with a clinical complete response (cCR) after neo-adjuvant chemoradiotherapy. Organ preservation as part of the standard clinical management for rectal cancer patients has since been validated in several international cohorts [4,5,6,7,8,9].

Unfortunately, only a limited number of patients qualify for organ preservation since only 10% of patients reach a pathological complete response (pCR) after short-course radiotherapy with delayed surgery [10,11], while 16% reach a pCR after long-course radiotherapy (LCRT) [12]. Since the tumor response to radiotherapy appears to be dose-dependent [13], dose escalation to the tumor is expected to lead to increased cCR and organ preservation rates [14,15]. Previous studies on dose escalation of LARC, summarized in a meta-analysis by Burbach et al. [14], show favorable tumor regression and pCR rates. The studies described had a boost dose of ≥ 60 Gy on the primary tumor. A more recent study, the RECTAL BOOST study [15], found that for a boost dose of 65 Gy to the primary tumor in LARC, near-complete or complete tumor regression was more common in the intervention group (69.4%) than in the control group (45.3%). However, they also found that the boost did not increase the pCR or sustained cCR rates. In the RECTAL BOOST study the majority of patients had a nodal stage of N1 or N2, which may explain why no pCR was achieved. This is underlined by other studies with node-positive patient cohorts that did not show sustained pCR or cCR rates [16,17]. We hypothesize based on these studies that, apart from increasing the boost dose, it is probably necessary to boost positive lymph nodes in order to achieve sustained cCR rates.

To this end we need a safe and efficient technique to increase the dose to the positive lymph nodes and the tumor. Escalation of the dose currently given to the full mesorectum will likely achieve this, but because of the large target volume, toxicity is also expected to increase. The other option is to separately boost the GTVs for the primary tumor and the suspected lymph nodes. Due to inter- and intrafraction anatomical variation, accurate dose coverage in this approach can only be achieved using adequate PTV margins. This limits the potential for dose escalation in rectal cancer. Moreover, when boosting both the primary tumor and lymph nodes within a single treatment plan, their relative motion might require larger treatment margins. Online adaptive MRI-guided radiotherapy offers good soft tissue contrast and the possibility to adapt the treatment to the daily anatomy. This approach has the potential to make dose escalation to multiple targets in rectal cancer feasible.

With online adaptive MRI-guided radiotherapy, daily plan adaptation can be performed through the use of two different strategies. One strategy, known as “adapt to shape” (ATS) [18], adapts the treatment plan every fraction using online re-delineation of all structures on the adaptation MRI (MRI_adapt_). The plan is then reoptimized, based on the adjusted contours. The other strategy, known as “adapt to position” (ATP), adapts the reference treatment plan based on a rigid translation.

In previous studies, the intrafraction motion and corresponding planning target volume (PTV) margins were determined for the primary tumor GTV [19,20,21,22]. These studies found margins between 6 mm and 12 mm. Mesorectal lymph node motion relative to the tumor, however, has not been studied before. The primary aim of this study, therefore, is to determine the margins required for GTV_ln_. The influence of the inter- and intrafraction motion on the treatment margin is different for the two adaptation methods. With ATS, GTV_prim_ and GTV_ln_ are redelineated separately on the daily MRI_adapt_ and the interfraction motion is effectively corrected by this redelineation. Here the only relevant motion is the intrafraction motion. For ATP, we performed a rigid registration of GTV_prim_, leaving an uncertainty in the position of GTV_ln_. With ATP, the interfraction motion of GTV_ln_ with respect to GTV_prim_ becomes relevant and is assumed to dominate the intrafraction motion.

The purpose of this study was to characterize the motion and define the required treatment margins of pathological mesorectal lymph nodes for two online adaptation strategies for sequential boosting. In addition, we determine the margins required for GTV_prim_ and study the effect of the anatomical location of GTV_ln_ on the margins.

## 2. Materials and Methods

### 2.1. Patients and MR-Linac Protocol

Data from 28 patients with intermediate-risk or locally advanced rectal cancer with one or more pathological mesorectal lymph nodes treated on a 1.5T MR-Linac (Unity, Elekta AB, Stockholm, Sweden) between October 2018 and May 2022 were analyzed. Patients were included in the Momentum registration study (NCT04075305) [23] and gave written informed consent for the use of their data. Patients received short-term radiotherapy (SCRT |5 fractions of 5 Gy) without dose escalation. An online adaptive workflow as previously described [22] was used using ATS. In short, pre-treatment a simulation CT and MRI were acquired on which the target volumes for elective treatment were delineated for plan optimization. Patients were advised to drink 250 mL water 30 min prior to simulation and the radiotherapy session on the MR-Linac to conform to the local bladder-filling protocol. On the day of treatment, four 3D T2-weighted MRI images were acquired: one for plan adaptation and three for verification prior to, during and post-treatment, respectively. The images acquired for adaptation (MRI_adapt_) and post-treatment verification (MRI_post_) from 5 fractions per patient were used in this study. Images of 5 daily fractions were used. The 3D T2-weighted MRI had a field of view (FOV) of 400 × 448 × 249 mm^3^, repetition time (TR) of 1300 ms, and echo time (TE) of 128 ms. MRI_adapt_ had a voxel size of 1.2 × 1.2 × 1.2 mm^3^ and acquisition time of 6 min, while MRI_post_ used 1.2 × 1.2 × 2.4 mm^3^ acquired in 3 min.

On the MR images, the primary gross tumor volume (GTV_prim_) and all suspected mesorectal lymph nodes (GTV_ln_) were delineated retrospectively using the contouring toolbox in Monaco v5.40.01 (Elekta, Stockholm, Sweden).

Mesorectal lymph nodes on MRI were classified as pathological based on the following criteria [24,25]: 1. A lymph node with a short-axis diameter ranging between 5 and 9 mm, combined with at least 2 of the following malignant morphological characteristics: a. a spiculated or indistinct border, b. heterogeneous texture, c. round shape; 2. a lymph node with a short-axis diameter <5 mm, combined with all three malignant morphological characteristics and 3. A lymph node with a short-axis diameter ≥ 9 mm.

Delineations on MRI_adapt_ were copied to the MRI_post_, and manually adjusted for each fraction. The anal verge was delineated on MRI_adapt_ of the first fraction. All scans of one patient were delineated by the same RTT and verified by a radiation oncologist with over 10 years’ experience.

### 2.2. Inter- and Intrafraction Displacement

#### 2.2.1. ATS: Intrafraction Displacement of GTV_ln_ and GTV_prim_

With the ATS strategy, both GTV_ln_ and GTV_prim_ are redelineated independently on daily MRI_adapt_ followed by plan reoptimization. This strategy corrects for the interfraction motion of both GTV_ln_ and GTV_prim_ independently, leaving the intrafraction motion of both structures between MRI_adapt_ and MRI_post_ as the primary remaining uncertainty. To determine the intrafraction displacement, the center of gravity (COG) of GTV_ln_ and GTV_prim_ were determined by uniformly sampling each volume with points and determining the average position of these points. For practical purposes this point can be interpreted as the center of the volume. Displacement of the GTV_prim_ and GTV_ln_ on MRI_post_ relative to MRI_adapt_ was characterized as the difference between the COGs and determined for each fraction in the left–right (LR), anterior–posterior (AP) and cranial–caudal (CC) direction. The effective intrafraction displacement during treatment was then estimated at ¾ of the total displacement, based on the approach in [26].

#### 2.2.2. ATP: Interfraction Displacement of GTV_ln_ with respect to GTV_prim_

With the ATP strategy, translations of a target are corrected each fraction through an isocenter shift on the pre-treatment CT. For this study, we assume a perfect match of GTV_prim_, thereby correcting all interfraction motion of GTV_prim._

Due to the relative motion of GTV_ln_ with respect to GTV_prim_, this potentially results in a discrepancy between the planned position and actual position of GTV_ln_ at the start of the treatment fraction. For this study, GTV_ln_ delineations were not available on the planning CT due to a lack of soft tissue contrast, influencing the accuracy of both GTV delineations. Therefore, we used the delineations on MRI_adapt_ of fraction 1 as a representative reference. We determined the COG of GTV_ln_ with respect to GTV_prim_ on MRI_adapt_ of fraction 1 for all patients. For subsequent fractions we assumed the COG of GTV_prim_ to be perfectly matched and determined the residual displacement of GTV_ln_ on these fractions with respect to its position on the reference fraction 1. As a result, interfraction motion was calculated on 4 fractions instead of 5.

### 2.3. Margin Calculation

We determined the margins for a hypothetical treatment where GTV_ln_ and GTV_prim_ and are boosted sequentially, directly following a homogeneous, elective irradiation of the mesorectum and lateral lymph node area. Given a group mean (GM), systematic error (Ʃ) and random error (σ), the PTV margin M_PTV_ was calculated using the Van Herk et al. margin recipe [27]:

$$M_{\mathrm{PTV}}=2.5\;Ʃ+1.64\;\left(\sqrt{\sigma^{2}+\sigma_{p}{}^{2}}-\sigma_{p}\right)+\mathrm{GM}$$

As we assume the boost to be given sequentially, σ*_p_* = 3.2 mm is used to describe the penumbra width in the pelvic area. A t-test was conducted to determine whether the GM significantly differed from zero (α = 0.05) and needed to be added to M_PTV_ to obtain asymmetrical margins.

For the ATS strategy, we determined GM, Ʃ, σ and M_PTV_ in the LR, AP and CC directions independently for GTV_prim._

If a patient has multiple lymph nodes, their individual motion patterns might not be independent and their motion with respect to GTV_prim_ is almost certainly correlated due to the movement of GTV_prim_ itself. Since the number of lymph nodes varied per patient from 1 to 4, this risks biasing the results due to clustering of the data. To avoid this, we resampled the original dataset 20 times, with each subset containing a single randomly selected lymph node for each patient. For each dataset we calculated the GM, Ʃ, σ and M_PTV_ for the LR, AP and CC directions independently. We then calculated the mean and standard deviation of the M_PTV_, GM, Ʃ and σ over all 20 resamplings. We did the same for the ATP strategy.

### 2.4. Relationship between Lymph Node Motion and Other Factors

Earlier studies on mesorectum shape variation [28,29] and tumor motion [20] suggest that the motion might be larger for target volumes in the upper anterior part of the mesorectum. Therefore, we also study the relationship between the magnitude of lymph node motion, the location in the mesorectum and the position relative to GTV_prim_ to determine whether location-specific treatment margins may be needed.

To study this, we determined (similar to [30]) for each lymph node on MRI_adapt_ of the first fraction: 1. the longitudinal COG-distance to GTV_prim_, 2. the longitudinal COG-distance to the anal verge and 3. the location within the mesorectum with respect to the mid-coronal plane of the mesorectum on each slide (anterior/posterior). Next, two mixed-effect linear regression models were employed (RStudio 2022.02.1 build 461) for the intrafraction motion of GTV_ln_ when using ATS and the interfraction motion of GTV_ln_ with respect to GTV_prim_ when using ATP with patient number and lymph node number as nested random effects and fractionation as a repeated measures variable. The dependent variable was the magnitude of the intrafraction motion of the lymph nodes and the magnitude of the interfraction motion of the lymph nodes with respect to GTV, respectively, calculated as the Euclidean distance of the directional displacements. Lymph node distance with respect to the anal verge, lymph node distance with respect to GTV_prim_ and lymph node location were set as covariates. The model was built including the fixed effects for the covariates, with an interaction effect between the repeated measures variable fraction and all covariates respectively and between distance to the anal verge and lymph node location with respect to the mesorectal coronal midline. The latter is based on the expectation that the effect of the distance to the anal verge on the magnitude of the displacement of the lymph node might also be influenced by its location in the axial plane, e.g., upper anterior lymph nodes might have a higher magnitude of displacement.

## 3. Results

### 3.1. Patient Characteristics

Patient and tumor characteristics are summarized in Table 1. For four out of 28 patients, one or more MRI_post_ were not available, resulting in a total of 135 fractions available for analysis. Of the 28 patients, eight had one suspected lymph node, 14 had two to three suspected lymph nodes and six had at least four suspected lymph nodes. The majority (61%) of primary tumors was located in the lower rectum, within 5 cm of the anal verge. A total of 84 pathological mesorectal lymph nodes were available for analysis with a median of three lymph nodes per patient. Most lymph nodes (74%) were located above the tumor and in the mid rectum, between 5 and 10 cm from the anal verge (62%).

### 3.2. Inter- and Intrafraction Displacement

The GM, Σ and σ of COG-displacement of GTV_ln_ and GTV_prim_ for intra- and interfraction displacement are shown in Table 2. Since we assumed GTV_prim_ to be perfectly matched every fraction no interfraction displacement of GTV_prim_ is reported.

#### 3.2.1. ATS: Intrafraction Displacement of GTV_ln_ and GTV_prim_

The group mean of the intrafraction COG-displacement of GTV_ln_ was significant (*p* < 0.05) in the cranial and posterior directions. This results in asymmetrical margins in the AP and CC directions. Σ and σ were similar in all directions.

**Figure 1:**
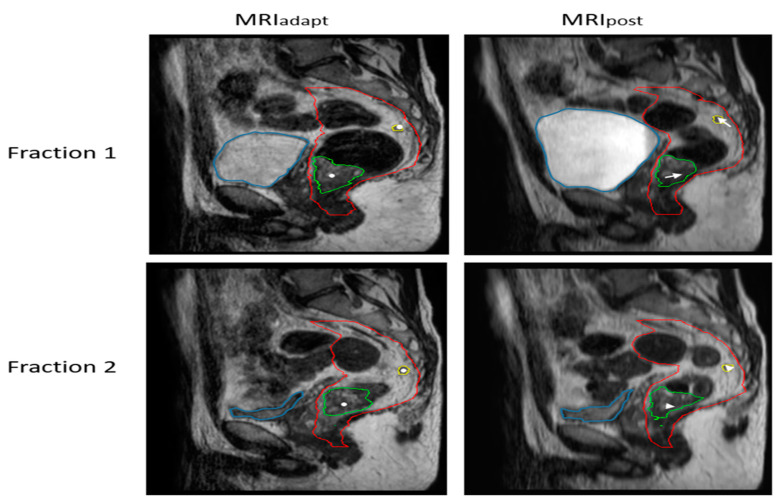
Magnitude of the intrafraction shift of GTV_prim_ (green contour) and GTV_ln_ (yellow contour) during two fractions as a result of bladder filling (blue contour) and consequent mesorectal shape variation (red contour). The white dots denote the COG of the structures on MRI_adapt_, and the arrows on MRI_post_ show the magnitude of the intrafraction shift. In fraction 1, as a result of increasing bladder filling, relatively large intrafraction motion is seen. In fraction 2, the bladder remains stable during treatment.

For GTV_prim_, the GM of AP was −1.2 mm and differed significantly from zero (*p* < 0.05). The smallest Σ was found in the LR direction, while σ was similar in all directions. Upon further examination of the MRI images, we observed a substantial intrafraction increase in bladder filling (Figure 1) for the majority of patients, which may explain the relatively large group mean displacement of GTV_prim_, whereas in other fractions the bladder remained stable during treatment. Since the majority of GTV_ln_ were located in the upper mesorectum proximal from GTV_prim,_ bladder filling resulted in a relatively large group mean displacement of GTV_ln_ in the cranial direction. In a small number of patients, a decrease in the rectal gas volume resulted in movement of GTV_prim_ and GTV_ln_ in the posterior direction.

The resulting margins for ATS were 3.5 mm (95% CI 3.3–3.7) LR, 4.5 mm (95% CI 4.3–4.7) cranial, 3.1 mm (95% CI 2.9–3.3) caudal, 5.1 mm (95% CI 5.0–5.3) anterior and 5.7 mm (95% CI 5.6–5.9) posterior for GTV_ln_ and 1.7 mm (LR), 4.7 mm (CC), 3.2 mm anterior and 5.6 mm posterior (AP) for GTV_prim_.

#### 3.2.2. ATP: Interfraction Displacement of GTV_ln_ with respect to GTV_prim_

The GM of interfraction displacement of GTV_ln_ with respect to GTV_prim_ was <1.0 mm in the LR and AP directions. The GM and Σ were largest in the CC direction and σ was similar in all directions. The estimated margins for ATP were 10.8 mm (95% CI 10.6–11.0) in the LR direction, 18.1 mm (95% CI 17.5–18.7) in the CC direction and 11.6 mm (95% CI 11.1–12.1) in AP.

### 3.3. Relationship between Lymph Node Motion and Other Factors

Using mixed-effect linear regression modeling, we found a significant but weak effect for distance from the anal verge and lymph node location on the intrafraction motion when corrected for repeated measures (shown in Table S1 in Supplementary Materials). In addition, after adjusting for the fixed effects, the estimates of variation in intercepts across lymph nodes and across patients were all significant (Table S2 in Supplementary Materials).

For interfraction motion with respect to GTV_prim_, no significant effects were found.

## 4. Discussion

The aim of this study was to characterize the motion and define the required treatment margins of the pathological mesorectal lymph nodes (GTV_ln_) and primary tumor (GTV_prim_) for two online MRI-guided adaptation strategies for sequential boosting of the GTVs.

The margins found for GTV_prim_ in this study are in line with earlier studies on intrafraction motion [20,21,22], except for Kleijnen et al. [19]. Kleijnen et al. [19] studied the motion as a function of time using repeated cine-MRI data and found a margin of 12 mm for the intrafraction motion, which is twice as large as the margins in the current study. The difference is possibly due to the difference in methodology: in their study margin calculation was based on a distance metric where 95% of the investigated time point voxels were required to fit within the margin in 90% of all fractions. This approach is more sensitive to individual outliers per fraction and therefore gives an overestimation of the margin in comparison with the van Herk approach, where coverage is evaluated for 90% of patients. Van den Ende et al. [20] reported PTV margins of 3.0 mm in the LR direction, 4.7 mm in the AP direction and 5.5 mm in the CC direction for intrafraction displacement of GTV_prim_. In Eijkelenkamp et al. [21] and Kensen et al. [22], isotropic margins 6.0 mm and 5.0 mm, respectively, were found. Based on the results of these studies and our study, a margin for GTV_prim_ of 5.0–6.0 mm isotropically appears adequate. In the case of anisotropic margins, the margins in the LR direction can be smaller (2.0–3.0 mm).

To date, the inter- or intrafraction motion of mesorectal lymph nodes and corresponding treatment margins has not been studied. Due to the different position of GTV_ln_ in the mesorectum, the margins differ compared to GTV_prim._ In this study we found that the group mean of the intrafraction motion of the GTV_ln_ was statistically significant in the cranial and posterior direction while this was the case in the posterior direction for GTV_prim._ The difference in group means is possibly due to increasing bladder filling and rectal filling during treatment. The majority (61%) of GTV_prim_ was located in the lower rectum and 89% of GTV_ln_ in the upper-mid rectum, cranial to the tumor. Increasing bladder filling during treatment caused the mesorectum to be pushed inwards at that level and consequently GTV_prim_ to be pushed towards the posterior side of the mesorectum, while GTV_ln_ were pushed towards the cranial and posterior directions. This is consistent with the findings of Van den Ende et al. [20], where most tumors were located in the upper- and mid-rectum and a group mean in the cranial direction was similar to GTV_ln_ in this study.

In this study, we compared two commonly used online adaptive MRI-guided strategies to determine which is suitable for dose-escalation purposes for GTV_ln_ and GTV_prim_. We showed that the smallest margins are found when performing daily redelineation and plan adaptation for each structure independently using an ATS strategy. With daily redelineation of each structure, only intrafractional differences in position need to be accounted for. Furthermore, we showed that the ATP strategy, using a match on GTV_prim_, results in a substantial uncertainty in lymph node position, requiring relatively large treatment margins. The reason for this is that the motion of GTV_ln_ and GTV_prim_ are largely independent. Based on this study, margins for GTV_ln_ of 3–4 mm (LR), 3–5 mm (CC) and 5–6 mm (AP) appear adequate. The smaller treatment margins make ATS the preferred strategy for irradiation of multiple structures with independent motion. ATP may be used for dose escalation to a single structure where only correcting translations is relevant.

We also built a linear mixed-effect model to assess whether the magnitude of lymph node motion correlates with factors such as the location in the mesorectum and the position relative to GTV_prim_ and the anal verge to determine whether location-specific treatment margins may be needed. We found a significant but weak effect of location-relation for both the intrafraction motion of GTV_ln_ and interfraction motion with respect to GTV_prim._ This is not in line with previous studies on motion patterns of GTV_prim_ [20] and mesorectum shape variation [28] that suggest location-relation. The weak effects found in this study might be explained by the fact that, despite their location, GTV_ln_ show similar motion.

A limitation of this study was that for the interfraction motion, MRI_adapt_ of fraction 1 was used a reference instead of the planning CT. Since the soft tissue contrast of CT is inferior to MRI, delineations of GTV_prim_ on the planning CT are not accurate and might have relatively large observer variability [31]. To avoid the bias of interobserver variability on our results, we therefore selected delineations on MRI adapt of fraction 1. Calculating the interfraction motion over four fractions instead of five might result in a slight overestimation of the systematic error and a slight underestimation of the random error, with a marginal effect on the final margin.

The factor of 2.5 in the margin recipe follows from the requirement that 90% of the targets be covered despite a systematic error. If this recipe is applied for multiple targets independently, this might imply that <90% of patients have both targets covered using this recipe, depending on the correlation in motion [32].

The treatment margins found in this study primarily account for uncertainties due to intra- and interfraction motion of the GTVs. In the total PTV used in clinical practice, other uncertainties such as delineation uncertainty, uncertainties in gantry positioning, MLC motion and image alignment should be included based on institutional practice. These uncertainties are, however, typically relatively small [33,34] and will have a marginal effect on the margins obtained.

This study only evaluated patients treated with SCRT with a hypothetical sequential boost. The motion and margins found here may not be appropriate for sequential boosting of long-course RT (LCRT) patients since the motion possibly differs during the last week of treatment. Previous studies showed a negative time trend in rectal volume in LCRT [35,36] as well as the incidence of diarrhea and bladder infections, which might result in smaller intrafraction motion. The outcomes of this study are therefore representative for SCRT patients.

## 5. Conclusions

Our study shows that ATP with a primary match on GTV_prim_ requires large PTV margins for the mesorectal lymph nodes. ATS results in the smallest margins and is therefore preferred for this purpose. With dose escalation of both primary tumor and involved lymph nodes, higher cCR rates may be achieved, resulting in increased organ preservation rates.

## Figures and Tables

**Table 1:** Patient characteristics. Data are displayed as numbers (%) unless indicated otherwise.

Patient Characteristics	n = 28 (%)
**Age in years, median (range)**	60 (52–67)
**Sex**	
Male	18 (64)
Female	10 (36)
**Tumor stage**	
cT2	4 (14)
cT3	21 (75)
cT4	3 (11)
**Nodal stage**	
cN1	23 (82)
cN2	5 (18)
**Tumor location (distance with respect to anal verge)**	
Lower rectum (0 to ≤5 cm)	17 (61)
Mid rectum (>5 to ≤10 cm)	11 (39)
Upper rectum (>10 cm)	-
**Number of lymph nodes**	84 (100)
Number of nodes per patient, median (range)	3 (1–4)
**Lymph node location with respect to tumor**	
Proximal	62 (74)
Peritumoral (at the same level of the tumor)	22 (26)
**Lymph node location with respect to mesorectum coronal midline**	
Anterior	38 (45)
Posterior	46 (55)
**Lymph node location with respect to anal verge**	
Lower rectum (0 to ≤5 cm)	9 (11)
Mid rectum (>5 to ≤10 cm)	52 (62)
Upper rectum (>10 cm)	23 (27)

**Table 2:** Summary information for GTV_ln_ (n = 30) and GTV_prim_ (n = 30) inter- and intrafraction motion calculated using van Herk PTV margin recipe. Translations are shown in left–right (LR), anterior–posterior (AP) and cranial–caudal (CC) directions. Positive values indicate motion in right, cranial, and posterior directions. GM = mean of patient motion, Σ = systematic error, σ = random error.

	Target Volume		LR (mm)	CC (mm)	AP (mm)
**Intrafraction displacement**	**GTV_ln_** (n = 28) **	GM	−0.2 (−0.3–−0.1)	0.7 (0.7–0.8) *	−0.3 (−0.4–−0.3) *
		Σ	1.2 (1.1–1.3)	1.2 (1.01–1.3)	1.9 (1.8–1.9)
		σ	1.4 (1.34–1.5)	1.9 (1.8–2.0)	1.7 (1.6–1.8)
		**M_PTV (ATS)_**	**3.5 (3.3–3.7)**	**3.1 (2.9–3.3|caudal)** **4.5 (4.3–4.7 |cranial)**	**5.1 (5.0–5.3 |anterior)** **5.7 (5.6–5.9 |posterior)**
	**GTV_prim_** **(n = 28)**	GM	0.0	0.2	−1.2 *
		Σ	0.6	1.7	1.6
		σ	1.0	1.4	1.3
		**M_PTV (ATS)_**	**1.7**	**4.7**	**3.2. (anterior)** **5.6 (posterior)**
**Interfraction displacement with respect to GTV_prim_**	**GTV_ln_** (n = 28) **	GM	0.5 (0.4–0.6)	0.8 (0.6–0.9)	−0.2 (−0.4–0.0)
		Σ	3.4 (3.3–3.5)	6.3 (6.0–6.6)	3.4 (3.1–3.6)
		σ	3.3 (3.2–3.4)	3.5 (3.4–3.6)	3.9 (3.8–4.0)
		**M_PTV (ATP)_**	**10.8 (10.6–11.0)**	**18.1 (17.5–18.7)**	**11.6 (11.1–12.1)**

* *p* < 0.001; ** Mean (95% CI) are reported based on 20 resampled datasets.

## Data Availability

The datasets generated and/or analyzed during the current study are not publicly available due to protection of individual patient privacy and the use of an in-house software but are available from the corresponding author on reasonable request.

## Supplementary Materials

The following supporting information can be downloaded at: https://www.mdpi.com/article/10.3390/cancers15041009/s1, Table S1: Linear mixed-effect model effect size estimates for the fixed effects on dependent variables intrafraction displacement of GTV_ln_ and interfraction displacement of GTV_ln_ with respect to GTV_prim_, Table S2: Variance attributed to the between-clusters and between-patients random effects.
