# Supplementary material for: Online Adaptive MRI-Guided Radiotherapy for Primary Tumor and Lymph Node Boosting in Rectal Cancer
Source: Cancers (Basel). 2023 Feb 5;15(4):1009. doi: 10.3390/cancers15041009 (PMC9953931; doi:10.3390/cancers15041009)
Supplement: Supplementary file 1 [file cancers-15-01009-s001.zip › cancers-2037727-supplementary.pdf]

Supplementary materials

Table S1: Linear mixed effect model effect size estimates for the fixed effects on dependent variables intrafraction displacement of GTV_{ln} and interfraction displacement of GTV_{ln} w.r.t. GTV_{prim}

Covariates	Dependent variable	
	**Intrafraction displacement of GTV_{ln} in mm (95% CI)**	**Interfraction displacement of GTV_{ln} w.r.t GTV_{prim} in mm (95% CI)**
**Constant**	4.0*** (2.2-5.9)	8.6** (2.6-14.5)
**Distance to anal verge**	<0.1 (-0.5-0.1)	<0.1 (-1.4-0.7)
**Distance to GTV_{prim}**	0.1 (-0.2-0.4)	-0.3 (-0.7-1.4)
**Position w.r.t. mesorectal mid coronal line posterior**	-1.3 (-3.0-0.3)	-0.9 (-5.4-3.6)
**Fraction**	<0.1 (-0.3-0.5)	-0.8 (-2.0-0.3)
**Distance to anal verge corrected for Fraction**	<0.1 (<0.1-0.4)	0.1 (<0.1-0.3)
**Distance to GTV_{prim} corrected for Fraction**	<0.1 (<0.1-0.1)	<0.1 (-0.3-0.2)
**Position w.r.t. mesorectal mid coronal line corrected for Fraction**	<0.1 (-0.3-0.2)	0.2 (-0.4-0.9)
**Position w.r.t anal verge and mesorectal mid coronal line**	0.2* (<-0.1-0.3)	<0.1 S(-0.4-0.6)
**Observations**	408	324
**Log Likelihood**	-814	-906
**AIC**	1653	1836
**BIC**	1701	1881

Note: *p<0.05; **p<0.01; ***p<0.001

Table S2: Variance attributed to the between clusters and between patients random effects

	Intrafraction displacement of GTV_{ln} (mm)			Interfraction displacement of GTV_{ln} w.r.t GTV_{prim} (mm)		
**Random effects**	**variance**	**95% CI**	**% of variance attributed to Random effect**	**variance**	**95% CI**	**% of variance attributed to Random effect**
**Nested (LN in patient)**	0.3	<0.1-0.7	8	3.1	0.9-5.9	9
**Patient**	0.7	0.2-1.4	20	20.6	10.4-35.9	60
**Residual**	2.6	2.2-2.9	72	10.6	8.8-12.7	31
**Total**	3.6			34.3		
